# Are Correlation Analyses Sufficient for Cardiac Output Method Comparison?

**DOI:** 10.1111/anec.70182

**Published:** 2026-03-26

**Authors:** Halit Emre Yalvaç, Bülent Görenek

**Affiliations:** ^1^ Department of Cardiology Eskisehir City Hospital Eskisehir Türkiye; ^2^ Department of Cardiology Eskisehir Osmangazi University Faculty of Medicine Eskisehir Türkiye

Dear Editor,

We read with great interest the recent study by Wang et al. evaluating the accuracy of three transthoracic echocardiographic techniques—fractional shortening, Simpson's method, and LVOT velocity–time integral (VTI)—for the assessment of cardiac output and stroke volume in hemodynamically unstable patients (Wang et al. 2026). The authors should be commended for addressing an important clinical question regarding noninvasive hemodynamic monitoring in critically ill patients. As the use of invasive monitoring techniques has declined due to potential complications and costs, bedside echocardiography has emerged as an attractive alternative for cardiac output assessment (Monnet and Teboul 2017; Messina et al. 2023).

Nevertheless, several methodological aspects of the study merit further consideration when interpreting the reported findings. First, the study population is relatively small. Only 12 patients were included, and the total of 54 measurements represents repeated observations within the same individuals. Although repeated measurements increase the number of data points, the effective sample size remains limited, which may influence the statistical robustness and generalizability of the results. This issue becomes particularly relevant when interpreting the very high correlation coefficients reported for the LVOT‐VTI method.

Second, the comparison between measurement techniques relied primarily on correlation analysis. While correlation coefficients describe the strength of association between two variables, they do not necessarily reflect agreement between measurement methods. In studies evaluating alternative hemodynamic monitoring techniques, agreement analyses—most commonly using Bland–Altman methodology—are generally considered more appropriate to determine whether two methods can be used interchangeably (Bland and Altman 1986). Incorporating such analyses could provide a clearer understanding of the actual agreement between transthoracic echocardiographic measurements and the reference technique.

Another point that warrants attention is the choice of reference method. In the present study, PiCCO monitoring was used as the comparator for echocardiographic measurements. Although PiCCO is widely used in critical care settings and has been validated in several clinical studies, it is not traditionally regarded as the definitive reference standard for cardiac output measurement, which has historically been pulmonary artery catheter thermodilution (Monnet and Teboul 2017). Consequently, the present comparison may be interpreted as an evaluation between two indirect measurement techniques rather than a strict validation against a gold‐standard method.

Finally, the included patient population appears clinically heterogeneous, including patients with sepsis, acute coronary syndromes, gastrointestinal bleeding, and malignancy‐related complications. These diverse pathophysiological conditions may influence cardiac performance and hemodynamic profiles differently, potentially affecting the accuracy of echocardiographic measurements. Future studies involving larger cohorts and more homogeneous patient populations may therefore provide additional insight into the clinical applicability of these techniques.

In conclusion, the study by Wang et al. provides valuable data regarding the potential role of transthoracic echocardiography in noninvasive hemodynamic assessment in critically ill patients. Further investigations with larger study populations and methodological approaches focusing on measurement agreement may help to better define the clinical role of echocardiographic techniques in cardiac output monitoring.

## Author Contributions

Both authors contributed to the conception, drafting, and critical revision of the manuscript and approved the final version.

## Conflicts of Interest

The authors declare no conflicts of interest.

## Data Availability

The authors have nothing to report.
